# Commercial Silver Diamine Fluoride (SDF) Products on Caries Lesion Progression in Primary Enamel: An *In Vitro* Study

**DOI:** 10.3290/j.ohpd.b871057

**Published:** 2020-12-14

**Authors:** Dayse A. Romão, Constanza E. Fernández, Lucineide de Melo Santos

**Affiliations:** a Assistant Professor, Faculty of Dentistry, Federal University of Alagoas, Maceió, AL, Brazil. Designed the experiments and the experimental design; performed the experiments; analysed and interpreted the data.; b Full-time Professor, School of Dentistry, University of Talca, Chile. Analysed and interpreted the data; drafted the paper; revised and approved the final version of the manuscript.; c Associate Professor, Faculty of Dentistry, Federal University of Alagoas, Maceió, AL, Brazil. Conceived the research idea; designed the experiments and the experimental design; revised and approved the final version of the manuscript.

**Keywords:** dental caries, primary teeth, remineralisation

## Abstract

**Purpose:**

Evidence has shown that silver diamine fluoride (SDF) at 30–38% has the potential to control and revert caries lesions. However, SDF can be found at lower concentrations on the market. In this study, we evaluated the effect of different commercially available SDF products on the progression of non-cavitated caries lesion in primary teeth assessed by a pH-cycling model.

**Materials and Methods: Subsuperficial caries lesions were formed in primary teeth using a demineralising solution for 96 h. Demineralised samples were randomly allocated to the following groups (n = 12/group):**

(G1) Negative Control, deionised water; (G2) Cariostatic, Inodon; 10%* SDF; (G3) Cariestop, Biodynamics, 12%* SDF; (G4) Cariostal, Iodontec, 16%* SDF; (G5) Cariestop, Biodynamics, 30%* SDF (*concentrations declared in the label). Products were applied according to the manufacturer’s recommendation and reapplied after 7 days. Samples were exposed to a pH-cycling model for 14 days. After the cycle was completed, samples were sectioned, analysed by polarised light microscopy, and lesion depth was estimated as indicator of caries lesion progression. Groups were compared by multiple comparisons test (p < 0.05).

**Results:**

The negative control group exhibited the greatest lesion depth. All SDF products reduced the caries lesions depth and differed from the negative control. It was a trend observed (G1>G2>G3>G4), but no statistical differences among G3, and G5, and between G4 and G5 were observed for lesion depth.

**Conclusion:**

The tested SDF products reduced the depth of non-cavitated carious lesions in primary enamel. Although SDF products with declared concentrations between 12% and 30% (G3, G4, and G5) demonstrated a similar lesion depth in primary enamel, the effect of the concentration remains unclear.

SDF has recently regained worldwide attention since their introduction on the US market.^16^ Despite the fact that the clinical applications of SDF include both management of dentine sensitivity and control of dental caries, SDF is a cost-effective preventive method used mainly to control caries lesions (at tooth level) in specific risk populations.^16^,^29^,^36^ The application of SDF once or twice a year can inhibit significantly active lesions and reduce the incidence of caries lesions.^5^,^14^,^31^ It has been shown to be safe in arresting cavities in preschool children^9^,^26^ and is also recommended to arrest cavitated lesions on permanent dentition.^32^ Thus, due to several practical advantages, such as reduced clinical time, low cost and ease of application, together with its clinical efficacy, SDF has gained support as a non-invasive therapy to treat caries lesions.^16^,^32^

Most of the evidence for SDF come from clinical studies using SDF at 30–38% in arresting dentine caries lesions,7–9,^20^,^27^,^40^; thus 30–38% SDF has been described as the most effective concentration.^5^,^14^,^36^ SDF has also been shown to prevent the progression of occlusal initial lesions when used at 38%^19^,^20^ or at 10%.^2^ Clinical studies that directly compared SDF at different concentrations showed that 38% SDF was more effective than 12% in arresting active cavitated caries in primary teeth.^12^,^13^,^38^ To the best of our knowledge and as stated by a recent clinical guideline,^36^ there is no clinical evidence of SDF for control enamel non-cavitated lesions. We did however find two registered clinical trials (NCT0147738523 and NCT02789202) that use SDF to arrest enamel caries lesions in primary teeth. However, until new evidence is available, the effect of SDF in non-cavitated enamel lesion remains unclear, as is the role of different SDF concentrations.

Since there are several commercially available products with different declared SDF concentrations, and there is a lack of SDF effect on non-cavitated enamel lesions, the aim of the current study was to evaluate the effect of different marketed products of SDF on the progression of non-cavitated caries lesions in primary enamel.

## Materials and Methods

### Experimental Design

This study was approved by the local research and ethics committee (No. 007629/2009-78).

Subsuperficial caries lesions were formed in primary human teeth and tooth samples were randomly allocated to five experimental groups (n = 12/group). Different commercial SDF products [claiming to have a specific SDF concentration (% declared on the label)] were assessed. The tested groups were: (G1) Negative Control, deionised water; (G2) Cariostatic, Inodon; 10% SDF; (G3) Cariestop, Biodynamics, 12% SDF; (G4) Cariostal, Iodontec, 16% SDF; (G5) Cariestop, Biodynamics, 30% SDF (Table 1). Products were applied according to the manufacturer's recommendation and reapplied after 7 days.^9^ Samples were exposed to a pH-cycling model for 14 days. After the cycle was completed, samples were sectioned, analysed by polarised light microscopy, and lesion depth was estimated as indicator of caries lesion progression. Groups were compared by multiple comparisons test (p < 0.05).

**Table 1:** Description of the experimental groups and manufacturer information.

Experimental groups	%SDF declared in the product label	Commercial name	Manufacturer	Application time recommended by the manufacturer (min)
G1	Negative control	‒ (deionised water)	‒	
G2	10%	Cariostatic	Inodon Porto Alegre, RS, Brazil	3
G3	12%	Cariestop	Biodynamics, Ibiporã, PR, Brazil	2–3
G4	16%	Cariostal	Iodontec, Porto Alegre, RS, Brazil	3
G5	30%	Cariestop	Biodynamics, Ibiporã, PR, Brazil	2–3

### Samples Selection and Caries Lesion Formation

Sixty primary canines without caries lesions, stains or any visible defects (by visual examination) were selected by using a stereomicroscope. The teeth were stored initially in thymol 0.1% to inhibit bacteria growth^32^ and at 4°C until use. A prophylaxis using pumice and water was performed on all teeth and subsequently isolated. To standardise the enamel surface area exposed to the treatments, an acid resistant varnish was applied to each tooth, leaving exposed an enamel area of 5 × 1 mm^15^ on the buccal surface.

Caries lesions were induced by immersing each tooth in 10 ml of a demineralising solution (2.2 mM CaCl_2_, 2.2 mM NaH_2_PO_4_, 0.05 M acetic acid with pH adjusted with 1 M KOH to 4.4) for 96 h.^17^ Subsuperficial lesions of 60–100 μm have been described to be formed with this methodology in primary human enamel.^17^ The teeth were washed in deionised water and divided randomly into five groups (n = 12/group). Samples were stored in 100% humidity until use.

### Treatments

Teeth were initially washed with deionised water and dried using absorbent paper. The exposed area of each sample was treated respectively with one of the following treatments (Table 1): (G1) Negative Control, deionised water; (G2) Cariostatic, Inodon; 10% SDF (concentration declared in the label); (G3) Cariestop, Biodynamics, 12% SDF; (G4) Cariostal, Iodontec, 16% SDF; (G5) Cariestop, Biodynamics, 30% SDF. One trained operator applied SDF on each sample using a cotton swab to distribute the SDF in the exposed as recommended by the manufacturers (Table 1). Then, SDF was allowed to remain in contact for 3 min for SDF to soak into and react with the lesion, followed by a 30 s rinse.^23^ Samples were retreated after 7 days^9^ of cycling model, as described earlier.

### pH-Cycling Model

Treated teeth were exposed to a pH-cycling model for 14 days.^17^ Daily, samples were immersed in a demineralising solution (described above) for 3 h at 37°C and then maintained in a remineralising solution (1.5 mM CaCl_2_, 0.9 mM NaH_2_PO_4_, 0.15 M KCl, with pH adjusted to 7.0) for 21 h at 37°C. The teeth were washed with deionised water and dried using paper before and after solution immersion. Fresh solutions were prepared for every cycle.^17^ The daily replacement of solutions was aimed to prevent mineral saturation and the accumulation of enamel dissolution products.

### Polarised Light Microscopy

After the complete period (14 days), each tooth was sectioned to obtain three samples of 100 µm thickness per tooth. The samples were obtained using a double diamond disc face 7020 (KG Sorensen, Barueri, SP, Brazil) coupled in a cutting machine. Each sample was polished with sandpaper grills at granulations of 300 to 600 micrometer (3M ESPE, Sumare, SP, Brazil). Before being analysed under the microscope, the samples were immersed in water for 24 h to complete filling of any spaces in the enamel. Subsequently, the enamel samples were fixed on glass slides to conduct polarised light microscopy analysis. A 10× lens was used to visualise the caries lesions and to determine the lesion depth. The lesion depth (LD) was determined in micrometers as the largest distance between the external enamel surface and the inner limit of the lesion. LD after 14 days was used as indicator of caries lesion progression. All measurements were performed by a single trained examiner.

### Statistical Analysis

LD values were compared among experimental groups. Normal distribution of errors and assumptions of homogeneity of variances were tested using the Shapiro Wilk test. Results were compared among groups by analysis of variance (ANOVA) followed by Tukey test (BioEstat, version 5.0, Belem, PA, Brazil). A 5% statistical significance level was set for all analysis.

## Results

**Fig 1:**
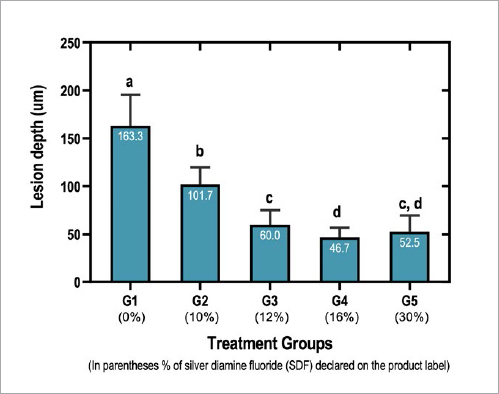
Depth of the enamel non-cavitated lesions (µm) according to the treatment groups (average ± SD; n = 12). In the negative control group (0% SDF), samples were treated with deionised water. Error bars represent standard deviations of the mean. Different letters represent statistical differences between the groups (p < 0.05, ANOVA/Tukey).

The depth of caries lesion ranged from 163.3 to 52.5 µm (Fig 1). The largest LD was observed in the negative control group (G1), and it was statistically different from all treated groups (p < 0.05). Data shows a trend on LD depending on the declared SDF concentration of commercially available products. However, no statistically significant differences were observed between the commercial products used in G3 and G5 (declared to contain 12% and 30% SDF, respectively) (p = 0.505), and between G4 and G5 (declared to contain 16% and 30% SDF, respectively) (p = 0.408) (Fig 1).

## Discussion

Our data suggest that all tested commercial SDF products were able to reduce caries lesion progression of non-cavitated enamel caries in primary teeth. A trend between declared SDF concentration and LD (indicator of caries lesion progression) was observed. However, there was a lack of statistical difference between products of G3 and G5, and between G4 and G5. Those groups G3, G4 and G5 declared to have SDF at 12%, 16% and 30% respectively. Considering that cavitated and non-cavitated lesions generally involve distinctive substrates, a different effect of SDF in non-cavitated enamel lesions versus cavitated lesions is expected. In fact, evidence has been consistent in showing that fluoridated therapies seem to be more effective in enamel than in dentine. For dentine usually bigger concentration, frequency or combination of methods is needed to achieve similar level of effectivity than in enamel.^10^,^11^,^35^ For SDF, an apparent dose-response effect has been observed when treating cavitated lesions,^12^,^13^,^38^ but not reported for non-cavitated lesions in enamel.

The mechanism of action effect of SDF in dental surfaces is still not fully understood.^25^ It is suggested that it is a combination of the formation of different mineral products plus an antimicrobial effect. Since the present study simulated non-cavitated lesions where oral biofilms have been removed, the reduction of LD is explained by the effect of mineral deposits on enamel surface by SDF and not by an effect in oral bacteria. It has been suggested that an insoluble protective layer is formed by different precipitates (ie, calcium fluoride-like products (CaF_2_), silver phosphate, silver chlorine,^31^,^39^ and fluorapatite (FAp).^25^ The layer formed for those precipitated seems to be responsible for the decrease of calcium and phosphorous loss from demineralised enamel and dentine,^39^ and consequently reducing caries lesion progression.

CaF_2_, a pH dependent and slow release reservoir of fluoride (F), is considered the putative mechanism of action of professionally applied F products.^30^,^34^ CaF_2_ provides fluoride to reduce subsequent demineralisation and promote remineralisation.^33^ Although we did not measure the actual CaF_2_ formation, it can be one of the reasons of the observed results. As described in Mei et al (2017),^25^ by 1972, Yamaga and colleagues suggested that CaF_2_ and silver phosphate were responsible for SDF anticaries effect.^25^ Nevertheless, it is still controversial that the primary effect of SDF is due to CaF_2_ formation because it seems to have a fast dissolution rate. It has also been pointed out that because of the alkaline nature of SDF, CaF_2_ formation can be limited; unlike acidulated F gel (which has an acidic pH and releases calcium during its application), SDF does not contribute to enamel dissolution during its application. Consequently, CaF_2_ is dependent on the availability of calcium from saliva or bacterial deposits.^24^ In our study, it is possible that SDF reacted with calcium and phosphate from Des-Re solutions precipitating CaF_2_ and/or fluorhydroxyapatite (FAp).^25^ In fact, in our study, demin and remin solutions were replaced daily in order to better simulated a clinical condition where saliva is constantly produced in order to keep the rate of CaF_2_ dissolution close to real levels. This daily replacement of solutions also aimed to prevent mineral saturation and the accumulation of enamel dissolution products.

As just mentioned, the precipitation of FAp is another explanation for the SDF effect observed in our samples. Fluoride available from SDF or from CaF_2_ formed by SDF can be a source for FAp precipitation during the Des-Re cycling model. Indeed, a recent *in vitro* study^25^ showed that SDF facilitated the formation of FAp and increased the size of crystals as the SDF concentration was increased.^25^ In this study, we have described SDF effect in terms of reduction of caries lesion progression (arrestment of the lesion) instead of remineralisation to describe SDF effect. This was taking in consideration that SDF is able to quickly harden dental surfaces. Besides FAp precipitation, other mineral layers (ie, containing silver phosphate)^31^ seem to play therapeutic roles by mechanically protecting tooth surface. However, this protective layer shields from further mineral loss but also from mineral deposition inside caries lesions, limiting lesion remineralisation.

Another point of discussion for this study is the actual need of using SDF to treat non-cavitated enamel caries lesions. Non-cavitated lesions can be arrested without SDF treatment^16^ by regular tooth brushing using a F toothpaste, rational sugar consumption and/or by applying fluoride varnishes.^14^,^18^,^22^ Although fluoride varnish (FV) is the recommended therapy to control non-cavitated proximal lesions,^32^ it has been suggested that SDF may have advantages over FV. SDF has the potential to immediately release most of the F contained in the product.^1^ In fact, it appears that SDF can arrest active caries lesions more effectively^31^ and faster than F varnish.^8^ Still, SDF can be used to treat active non-cavitated lesions in communities where resources and dental care services are limited and/or lack of patient compliance. In those particular cases, and preferably in non-aesthetic areas as posterior interproximal surfaces or pit and fissures, SDF can be an alternative to quickly arrest enamel lesions in primary dentition. Because of staining risk, SDF require a targeted application (ie, SDF should be site-specifically applied different to FV that could be applied in all dental surfaces).^1^ In fact, our demineralised primary enamel samples exhibited discoloration from grey to black due to enamel porosity. According to a recent *in vitro* study, no statistically significant differences on staining are expected between SDF at 38% and 12%.^28^ Despite the fact that tooth staining after SDF application is a disadvantage of the therapy, most parents have accepted discoloration in primary dentition,^4^,^6^ and its benefits appear to be much larger than its aesthetic disadvantage.^3^,^16^,^21^,^29^

Regarding methodological aspects, the use of natural teeth and the maintenance of the intact enamel surfaces are the strengths of the current study. Although the pH-cycling model used as a reference for our study^17^ appears to have certain validity (dose-response), the use of published formally validated pH-cycling models for primary teeth^37^ can be ideal to verify our findings. Because SDF also have been described as a bactericidal and bacteriostatic agent,^39^ it appears reasonable that future investigations study SDF effect by using models that includes oral biofilms. Such models can simultaneously evaluate the effect of SDF on bacteria and mineral exchange. It is also important to mention that commercial products may not contain the expected F content.

## Conclusion

Future studies testing comparable products at different concentrations, and/or using relevant models are needed to confirm our findings about SDF controlling enamel caries lesion progression.

Despite the limitations of the study, our results suggest that application of commercial SDF products, regardless of its declared SDF concentration (12–30%), may reduce the LD of non-cavitated enamel caries in primary teeth.
